# Time to scale up molecular surveillance for anti-malarial drug resistance in sub-saharan Africa

**DOI:** 10.1186/s12936-021-03942-5

**Published:** 2021-10-13

**Authors:** Christian Nsanzabana

**Affiliations:** 1 Department of Medicine, Swiss Tropical and Public Health Institute, Socinstrasse 57, 4002 Basel, Switzerland; 2 University of Basel, P.O. Box, 4003 Basel, Switzerland

## Abstract

Artemisinin resistance has emerged and spread in the Greater Mekong Sub-region (GMS), followed by artemisinin-based combination therapy failure, due to both artemisinin and partner drug resistance. More worrying, artemisinin resistance has been recently reported and confirmed in Rwanda. Therefore, there is an urgent need to strengthen surveillance systems beyond the GMS to track the emergence or spread of artemisinin and partner drug resistance in other endemic settings. Currently, anti-malarial drug efficacy is monitored primarily through therapeutic efficacy studies (TES). Even though essential for anti-malarial drug policy change, these studies are difficult to conduct, expensive, and may not detect the early emergence of resistance. Additionally, results from TES may take years to be available to the stakeholders, jeopardizing their usefulness. Molecular markers are additional and useful tools to monitor anti-malarial drug resistance, as samples collected on dried blood spots are sufficient to monitor known and validated molecular markers of resistance, and could help detecting and monitoring the early emergence of resistance. However, molecular markers are not monitored systematically by national malaria control programmes, and are often assessed in research studies, but not in routine surveillance. The implementation of molecular markers as a routine tool for anti-malarial drug resistance surveillance could greatly improve surveillance of anti-malarial drug efficacy, making it possible to detect resistance before it translates to treatment failures. When possible, ex vivo assays should be included as their data could be useful complementary, especially when no molecular markers are validated.

## Background

The development of resistance to the currently used anti-malarial drugs is threatening the major gains in malaria control and elimination made over the last decade. Artemisinin resistance, defined as delayed parasite clearance following treatment with artemisinin monotherapies or artemisinin-based combination therapy (ACT), has been associated with specific mutations in the *Plasmodium falciparum kelch 13* gene (*Pfk13*) [1]. Those validated molecular markers were initially observed in the Greater Mekong Sub-region (GMS), followed by ACT failures due to both artemisinin and partner drug resistance [2–9]. The recent reports of a validated molecular marker of artemisinin resistance in Rwanda [10, 11], and its association with delayed parasite clearance [12], are a major threat to malaria control and elimination in sub-Saharan Africa. Even though the continuous high efficacy of the first- and second-line anti-malarial drugs (artemether-lumefantrine and dihydroartemisinin-piperaquine) in Rwanda is reassuring, an improved scheme for monitoring anti-malarial drug resistance is warranted to mitigate the spread of artemisinin resistance, and ACT failure. Currently, the World Health Organization (WHO) recommends therapeutic efficacy studies (TES) for monitoring drug efficacy and resistance [13], whereas molecular markers and ex vivo monitoring are optional. There is no doubt that anti-malarial drug policy change should be based on TES results; however, molecular and ex vivo data have played an important role in confirming and monitoring artemisinin and partner drug resistance in the GMS [3, 9, 14, 15]. Indeed, partial resistance to artemisinin is difficult to assess in vivo, especially in high transmission settings where acquired immunity is a major confounding factor [16–18]. Moreover, TES are time and resources consuming, and may be difficult to conduct in low transmission settings, where the risk of de novo emergence of resistance is highest [19–21], due to the low number of patients.

### Molecular markers as early warning tools

Molecular markers offer an additional strategy to monitor the early emergence and spread of anti-malarial drug resistance, are not impacted by host immunity, and may be more cost effective when implemented for routine surveillance. Retrospectively, it has been suggested that partial sulfadoxine-pyrimethamine resistance had multiple origins including areas of high transmission in Eastern Africa [22, 23] and, interestingly, the same region seems to be a hotspot for partial artemisinin resistance [12, 24]. Today, tools (molecular markers) to closely monitor the early emergence and spread of artemisinin and partner drug resistance are available. This knowledge should be used to establish a comprehensive molecular surveillance system to avoid the mistakes from the past, when the spread of resistance to anti-malarial monotherapies has been detected at a late stage, contributing to thousands of deaths in the meantime. Molecular markers cannot predict treatment outcome at an individual level, however their increase often precedes that of treatment failures [25]. While monitoring *Pfk13* mutations is of paramount importance, monitoring partner drug resistance molecular markers is also crucial [26]. Indeed, high prevalence of *Pfk13* validated molecular markers is not usually associated with treatment failure [27], as evidenced by recent data from Rwanda where the efficacy of both first and second-line treatments is still high despite the increasing prevalence of the *Pfk13* 561 H mutation (Table 1). Currently, molecular surveillance is often done retrospectively based on convenience sampling, and does not provide an accurate estimation of resistance on a national or sub-regional level. For example, when looking at available molecular data for Rwanda, there are large spatiotemporal gaps [10–12, 28], and no clear trend is discernible (Table 1). However, high prevalence of the confirmed artemisinin resistance marker 561 H at two sites (Masaka and Rukara) are worrisome, especially as samples have been collected in 2018, and the current situation could be worse. Moreover, the prevalence of this marker has increased from 7 to 20% in Masaka between 2015 and 2018 (Table 1), even though the small sample size does not allow for definitive conclusions, but it is likely that the prevalence is even higher now, and only routine molecular monitoring could accurately assess the trend.

**Table 1:**
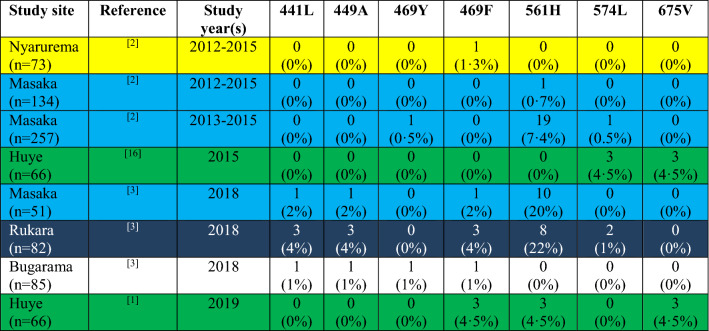
Prevalence of * PfKelch13 * mutations associated with artemisinin resistance in five different sites in Rwanda from 2012 to 2019

Each colour represents data from the same sites

### Molecular routine surveillance to inform TES

Molecular routine surveillance should not only be conducted in a few sentinel sites, but rather on a large network of health facilities to capture the complex spatial dynamics of evolving resistance [29]. Samples collected from patients attending selected health facilities in the different regions of a country, should be analysed on a regular basis and generated data used to map the spatiotemporal dynamics of molecular markers of interest [29–32]. The data could be then used to inform and calibrate mathematical models of malaria transmission aiming at guiding interventions strategies, for example to select the sites for TES, using specific thresholds for artemisinin and partner drug resistance markers prevalence, to assess either delayed parasite clearance or treatment failure, respectively [21, 33]. Indeed, it is difficult to predict where resistance will emerge, and a more flexible scheme with rotating sites for TES based on molecular markers prevalence and models prediction may be more appropriate for early detection of resistance. Mathematical models could be used as well to predict when treatment failure could occur based on the molecular markers prevalence, giving more time for policymakers to prepare the change in anti-malarial drug treatment policy [34, 35].

**Fig. 1:**
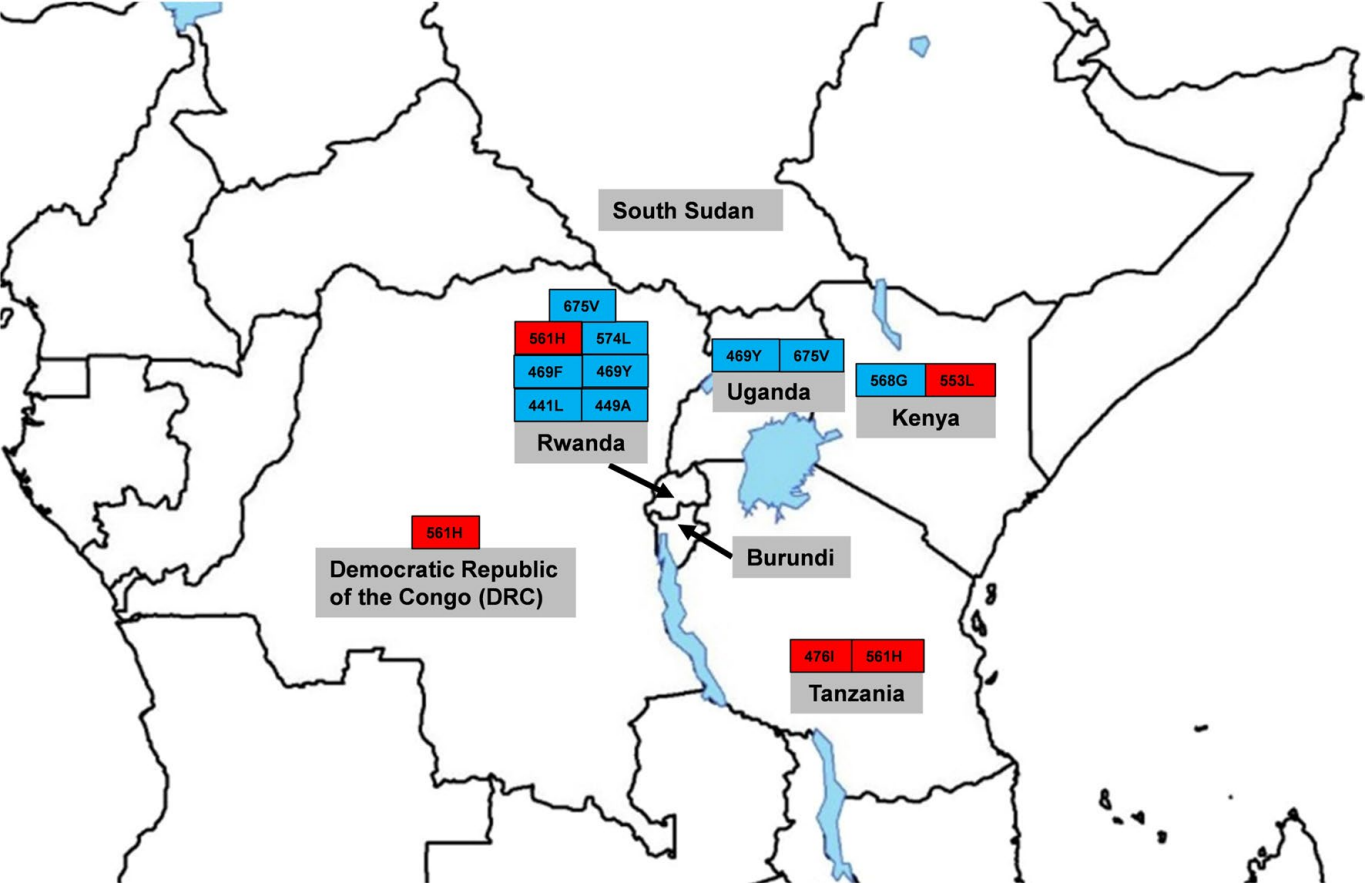
Map showing countries with WHO validated (in red) and candidate (in blue) artemisinin resistance markers in the Great Lakes region. (adapted from [11, 12, 24])

Logistically, sample collection would require only dried blood spots collection at selected health centres when patients have a confirmed malaria diagnosis. The molecular analysis could be centralized at regional, national or sub-regional laboratory to maximize the cost effectiveness of the surveillance system [36]. With the increasing availability of high throughput techniques and assays for molecular markers of resistance genotyping in malaria endemic countries, there is an opportunity to strengthen the capacity of National Malaria Control Programmes (NMCPs) for molecular monitoring of anti-malarial drug resistance [37, 38]. Cross-border collaboration is also critical, especially in regions such as the Great Lakes (Fig. 1), where validated resistance markers have been detected in different countries, including Rwanda, Democratic Republic of the Congo, Kenya, and Tanzania (Fig. 1). Regional monitoring is key to track resistance, as parasites will spread quickly from one country to another. The establishment of regional reference laboratories associated with regional data repositories could facilitate the prompt detection of resistance and early implementation of mitigation strategies; and the malaria community must leverage on the different initiatives on the continent to improve access to the infrastructure and technical expertise for high throughput molecular analyses [39].

### Ex vivo assays: a useful, but difficult tool to implement

Ex vivo assays have played an important role in monitoring artemisinin resistance in the GMS [3, 15, 40, 41]. Even though they are often used for phenotypic assays to validate molecular markers of resistance, where available they can be useful to monitor resistance. Indeed, as for molecular markers, immunity is not a confounding factor for ex vivo assays, even though parasite culture may reduce the complexity of the infection by preferentially selecting specific clones. However, ex vivo assays can be a valuable tool for drugs with no validated molecular markers, such as lumefantrine and pyronaridine, the former being the partner drug of the most widely used ACT, and the latter, the most recent ACT partner drug approved by the WHO. Moreover, compared to molecular markers, ex vivo assays are difficult to implement, as fresh blood is required for parasite culture and the high intra and inter-assays variability limits their ability for spatiotemporal dynamics assessment [42].

## Conclusions

Anti-malarial drug resistance is a serious threat to malaria control and elimination, and resistance monitoring is crucial to maintain the high efficacy of the current anti-malarial drugs. Anti-malarial drug efficacy monitoring schemes should take the full advantage of molecular and ex vivo culture techniques, as they may be the most appropriate tools to provide early warning signals of anti-malarial drug resistance in high transmission settings. Reinforcing routine molecular surveillance programme could help detecting the emergence and spread of artemisinin and partner drug resistance at an earlier stage, before it translates to treatment failures.

## Data Availability

Not applicable.

## Abbreviations

ACT: Artemisinin-based combination therapy
GMS: Greater Mekong Sub-region
NMCP: National Malaria Control Programme
TES: Therapeutic efficacy study
WHO: World Health Organization
